# Quantum Critical Behavior in a Concentrated Ternary Solid Solution

**DOI:** 10.1038/srep26179

**Published:** 2016-05-18

**Authors:** Brian C. Sales, Ke Jin, Hongbin Bei, G. Malcolm Stocks, German D. Samolyuk, Andrew F. May, Michael A. McGuire

**Affiliations:** 1 Materials Science and Technology Division, Oak Ridge National Laboratory, Oak Ridge, Tennessee, USA 37831-6056.

## Abstract

The face centered cubic (fcc) alloy NiCoCr_x_ with x ≈ 1 is found to be close to the Cr concentration where the ferromagnetic transition temperature, T_c_, goes to 0. Near this composition these alloys exhibit a resistivity linear in temperature to 2 K, a linear magnetoresistance, an excess –TlnT (or power law) contribution to the low temperature heat capacity, and excess low temperature entropy. All of the low temperature electrical, magnetic and thermodynamic properties of the alloys with compositions near x ≈ 1 are not typical of a Fermi liquid and suggest strong magnetic fluctuations associated with a quantum critical region. The limit of extreme chemical disorder in this simple fcc material thus provides a novel and unique platform to study quantum critical behavior in a highly tunable system.

Quantum critical behavior has been associated with some of the most exotic emergent states of matter including high-temperature superconductivity^1^,^2^. Much of the research into quantum critical point (QCP) physics has been hampered by the lack of model systems simple enough to be analyzed by theory^3^. Here, we show that the concentrated solid solution fcc alloys, including the so-called high-entropy alloys, are *ideal model systems* to study the effects of chemical disorder on emergent properties near a quantum critical region.

The recent synthesis of single-phase concentrated solid solutions has provided new frontiers for material physics research^4^,^5^. These alloys exhibit exceptional mechanical properties as compared to conventional alloys^6^ and exhibit enhanced resistance to radiation damage^7^. The alloys are characterized by a simple face-centered cubic (fcc) or body-centered cubic crystal structure (bcc) and two or more principle elements. If the alloys have five or more elements in equal atomic ratios (e.g. NiCoCrFeMn), they are often referred to as high-entropy alloys^7^. Most traditional multielement alloys have multiple phases and complex microstructures, or often have a majority element that can be considered the solvent and minority elements as dopants or solutes. In single-phase concentrated solid solutions, however, the elements randomly occupy an ordered fcc or bcc lattice with all elements having roughly equal concentrations. This results in locally disordered chemical environments, unique site-to-site distortions, and extreme complexity at the electron level. In spite of the inherent chemical disorder, these alloys are single phase with a cubic crystal structure and can be prepared as large single crystals. The alloys thus represent an interesting class of model materials for investigating the effects of a particular type of disorder on the cooperative response of the electrons, spins and lattice.

During our investigation^8^ of the basic electrical, magnetic, and thermal transport properties of a series of eight fcc Ni-based single-phase single-crystal concentrated alloys (Ni, NiCo, NiFe, NiFeCo, NiCoCr, NiCoCrFe, NiCoCrFeMn, NiCoCrFePd), we noticed that the low temperature resistivity of the NiCoCr alloy was linear in temperature at least down to our base temperature of 2 K. This is highly unusual for a metallic alloy in this temperature regime and was not observed for any of the other Ni-based alloys, which exhibited a resistivity that followed the expected T^2^ or T^5^ behavior^8^. A linear resistivity for a metal at low temperatures, although not fully understood, is often an indication of the proximity to an unusual quantum state associated with a quantum critical point (QCP)^9^. As shown below this is indeed the case for NiCoCr_x_ which is close to a ferromagnetic QCP for x ≈ 1. Simple fcc single-phase alloys with compositions near NiCoCr thus appear to be model systems for investigating how chemical disorder affects the cooperative response of the charge, spin, and lattice degrees of freedom near a QCP (or a quantum critical region as is the case for systems with significant disorder)^10^,^11^. Characterizing and understanding the physical signatures of QCP behavior in this new class of alloys provides the motivation for the present work. The concentrated NiCoCr_x_ alloys share some features of quantum critical behavior with other systems at low temperatures, such as a linear resistivity at very low temperatures (2K), an additional –TlnT or power law contribution to the low temperature heat capacity, and a linear magnetoresistance. However, an exponential variation of the dc magnetic response with concentration was observed, which was totally unexpected.

## Results and Discussion

**Figure 1:**
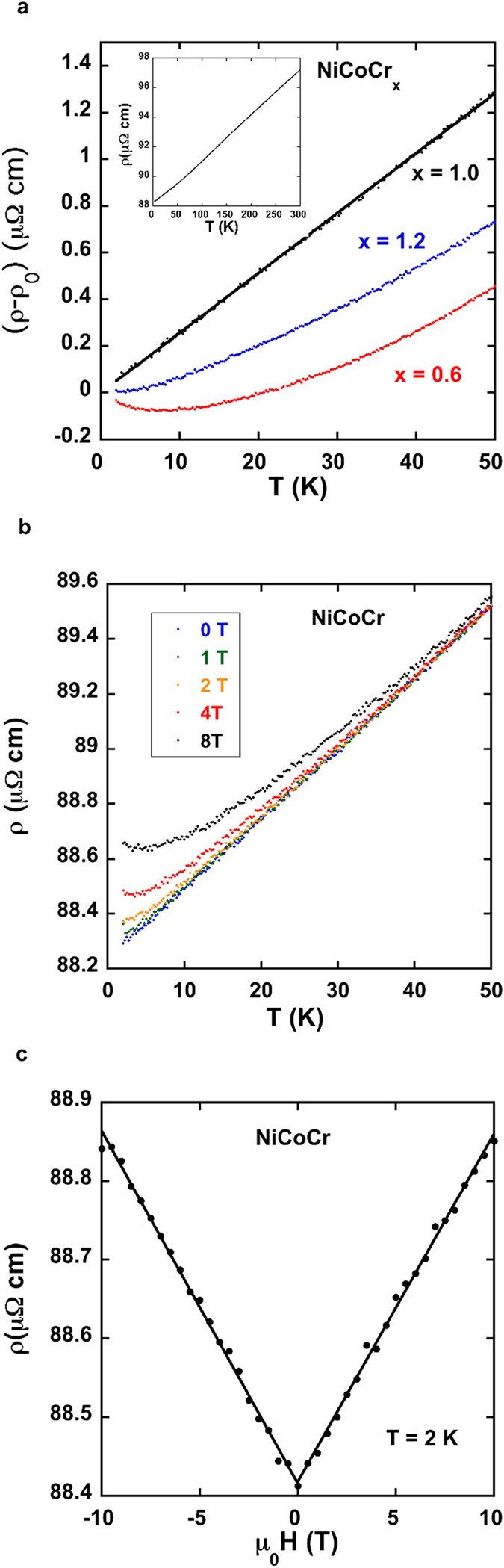
Linear Resistivity and Magnetoresitance. (**a**) Resistivity, ρ, of NiCoCr versus temperature from 2 K to 300 K (inset). The main panel shows the linearity of (ρ-ρ_0_) for NiCoCr versus temperature, where ρ_0_ is the residual resistivity. A linear fit to through the (ρ-ρ_0_) data is also shown. Also shown in main panel is (ρ-ρ_0_) versus temperature for two compositions away from the critical region. NiCoCr_1.2_ is a Pauli paramagnet, and NiCoCr_0.6_ is a ferromagnet with T_c_ ≈ 75 K. For both compositions the temperature dependence of the resistivity deviates strongly from linear. The increase in the resistivity below 5 K for NiCoCr_0.6_ may be due to the Kondo effect but is more likely due to the effects of electron-electron scattering in a disordered system, which can produce similar behavior^32^,^33^. (**b**) Resistivity of NiCoCr versus temperature for applied magnetic fields of μ_0_H = 0, 1,2,4 and 8 Tesla. (**c**) Magnetoresistance of NiCoCr at 2 K illustrating the linear dependence on magnetic field. Since all of the NiCoCr_x_ alloys (including x = 0) exhibit a similar linear magnetoresistance (see Supplemental section), this behavior is not associated with the quantum critical region and is likely directly related to the effects of disorder.

The resistivity of NiCoCr from 2–300 K is shown in the inset of Fig. 1. The residual resistivity, ρ_0_, is high in this family of concentrated alloys, typically in the range of 90 μΩ-cm. This high value can be understood quantitatively using the *ab initio* Korringa-Kohn-Rostoker Coherent-Potential-Approximation (KKR-CPA) theoretical formalism as has been explained previously^7^,^8^ and is briefly discussed again in the supplemental information. The addition of Cr to a ferromagnetic NiCo alloy disrupts electron transport in both the minority and majority spin channels, resulting in a rapid increase in the residual resistivity. This formalism also nicely explains the rapid decrease in the Seebeck coefficient from about 30 μV/K for NiCo at 300 K to 0.9 μV/K for NiCoCr. The addition of Cr also leads to a type of frustration since the spins on the Cr atoms want to be antiparallel to neighboring Cr atoms and to the Ni and Co spins, which cannot be perfectly satisfied in a random solid solution on an fcc lattice. In insulating spin systems frustration can lead to a spin liquid that is characterized by strong quantum fluctuations and no magnetic order to T = 0^12^. The main panel of Fig. 1. illustrates the linearity of the resistivity of NiCoCr between 2 and 50 K, which is very unusual for a metal in this temperature range^9^. For NiCoCr_x_ alloys well away from x = 1, a more normal metallic resistivity is recovered (Fig. 1). For values of x between about 0.8 and 1.0 the resistivity exhibits a nearly linear resistivity to 2 K, but for x = 1.1 and x = 0.7, the resistivity is more normal. (See Supplemental Fig. S4).

The response of the resistivity of NiCoCr to a magnetic field is shown in Fig. 1b. As the magnetic field is increased, the low temperature linear resistivity is lost (Fig. 1b) and the temperature dependence resembles a normal metal. The data look qualitatively similar to what occurs in Sr_3_Ru_2_O_7_, except for that compound the resistivity is linear with an applied field μ_0_H = 7.9 T, and more “normal” at other magnetic fields (including H = 0)^9^. The physics of Sr_3_Ru_2_O_7_ however, is clearly different from the present alloys, which are chemically highly disordered. Qualitatively, the effect of a magnetic field on the resistivity of NiCoCr, is similar to the effect of reducing x. (Compare Fig. 1b., 8T data with Fig. 1a, x = 0.6) although there is no simple scaling relationship.

The low temperature magnetoresistance, although small in magnitude (≈0.5% at 10 T) is remarkably linear at 2 K with no evidence of saturation (Fig. 1c). A linear magnetoresistance is found in graphene, topological insulators, and some other quasi-2D bulk materials where it is associated with linear bands near the Fermi energy and Dirac fermions and nodes^13^,^14^. However a linear magnetoresistance can also occur in simple metals such as potassium and copper^15^,^16^. For NiCoCr, the origin of the linear transverse magnetoresistance is not clear although the combination of a high residual resistivity and metallic carrier concentration (≈9 × 10^22^ electrons/cm^3^ estimated from Hall data- see supplemental) should result in a small magnetoresistance, as is observed^16^. In addition, a linear positive transverse magnetoresistance is observed at 2 K for all of the NiCoCr_x_ alloys (including x = 0), which suggests it is related to common features of the electronic band structure and chemical disorder (see supplemental) and not related to the quantum critical region.

**Figure 2:**
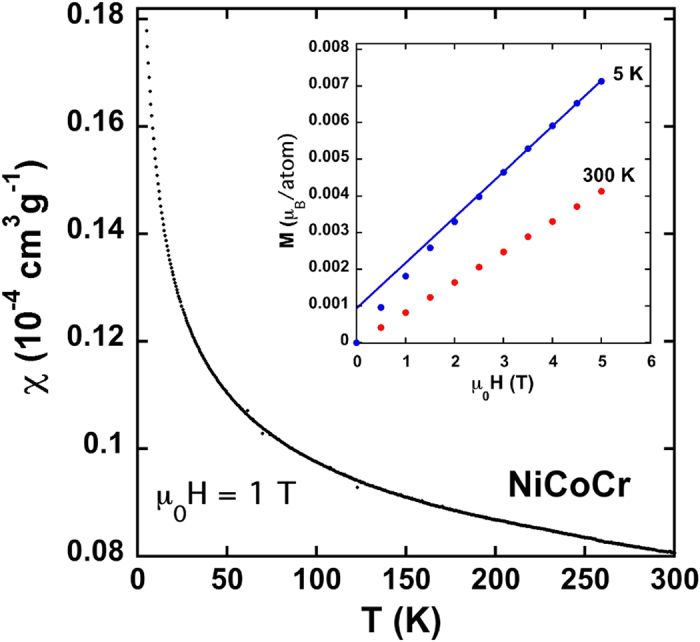
Magnetic susceptibility versus temperature for NiCoCr with an applied magnetic field of 1 Tesla. There is no evidence of magnetic order and the magnitude of the susceptibility is similar to that of Pd metal. Magnetization curves are linear at 300 K but show a small amount of curvature at 5 K (Inset).

The magnetic susceptibility and magnetization data for NiCoCr are shown in Fig. 2. The susceptibility data are best described as an enhanced Pauli paramagnet, similar in magnitude to Pd metal^17^, with no evidence of magnetic order down to 2 K. The magnetization curve at 300 K is linear up to our highest measuring field (5 Tesla), but at 5 K there is a small amount of curvature (Fig. 2 inset). The extrapolation of a linear fit to the high field magnetization data (3–5 Tesla) to μ_0_H = 0 yields a moment of 0.001 μ_B_/atom. If NiCoCr were ferromagnetic, this would be an estimate of the spontaneous moment, M_0_.

The origin of the unusual properties exhibited by NiCoCr is investigated in a series of alloys, NiCoCr_x_, where the Cr concentration is varied with 0 < × < 1.2. All of the alloys have the same simple fcc structure with the three elements randomly distributed on the fcc lattice (see supplemental section for more details).

**Figure 3:**
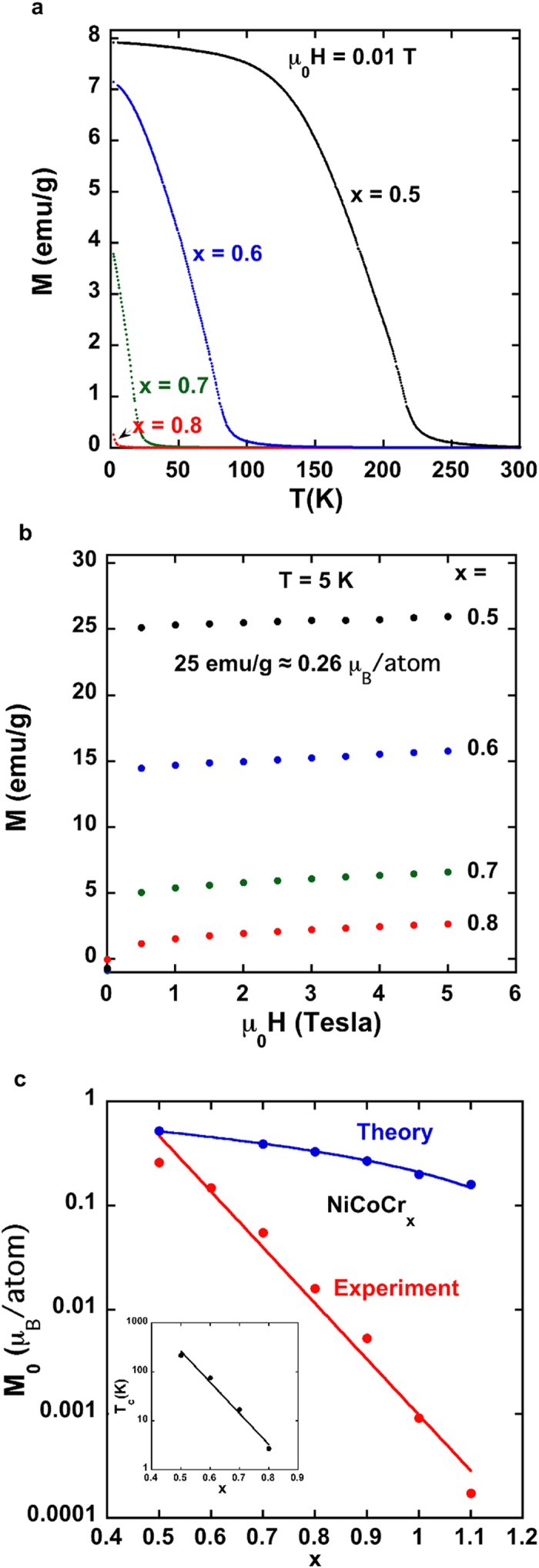
Magnetization data-experiment and theory. (**a**) Magnetization versus temperature in a small applied magnetic field of 0.01 T for four NiCoCr_x_ alloys. (**b**) Magnetization vs magnetic field at T = 5 K for the same four alloys. (**c**) Estimate of the spontaneous magnetic moment versus Cr concentration from magnetization curves at 5 K (see Figs 2 and 3b). M_0_ is determined from linear fits to magnetization curve data for magnetic fields between 3 and 5 Tesla extrapolated back to μ_0_H = 0 (see Fig. 2 inset). The red line is an exponential fit to the M_0_ data [M_0_ = 226 exp(−12.36 x)]. The inset shows the dependence of T_c_ on x, which has a similar exponential dependence [T_c_ = 4.0019 × 10^5^ exp(−14.64 x)]. From KKR CPA calculations, the average spontaneous moment per atom should vary linearly with x. The blue line is a linear fit to the calculated values for M_0_. For x = 0 (not shown) and 0.5, which is far away from the critical Cr concentration region of x_c_ ≈ 0.8–1, the theoretical value of M_0_ is close to the measured value, but the values diverge as x_c_ is approached. For x = 0, the experimental and theoretical values for M_0_ are 1.34 and 1.11 μ_B_/atom respectively.

The evolution of ferromagnetism in alloys with reduced Cr concentration is particularly striking (Fig. 3). Both the ferromagnetic transition temperature, T_c_, and the low temperature spontaneous moment per atom, M_0_, change exponentially with x. The estimated Curie temperatures obtained from Fig. 3 (and Arrott plots- see supplemental) are 212, 77, 20, and 2.5 K for x = 0.5, 0.6, 0.7 and 0.8 respectively. The remarkably rapid variation of T_c_ and M_0_ with x was not expected by a KKR-CPA mean field theory calculation (Fig. 3c) or by comparing the NiCoCr_x_ to other magnetic QCP systems such as Ni_x_Pd_1−x_^18^, Cr_1−x_V_x_^19^, Ni_1−x_Cr_x_^20^ where the concentration dependence of T_c_ on x is usually linear or close to linear. The strong and rapid suppression of ferromagnetism for 0.5 < × < 1.0 suggests that unusually strong magnetic fluctuations and frustration prevent magnetic order. In addition, the small values of the average magnetic moment per atom (Fig. 3) should make these alloys more susceptible to quantum effects^12^. One interesting example of a rapid, almost exponential decrease of the magnetic ordering temperature with doping occurs in the hole doping of a Mott insulator such as La_2-x_Sr_x_CuO_4_^21^. The rapid decrease of the Neel temperature with Sr doping is believed to arise by the breaking of antiferromagnetic exchange paths and the simultaneous introduction of additional ferromagnetic exchange paths. In the NiCoCr_x_ alloys there are also competing ferromagnetic and antiferromagnetic interactions that will rapidly change with Cr doping, and hence there is some analogy with the hole-doped cuprates. However the analogy is not perfect since similar competing ferromagnetic and antiferromagnet interactions occur in Ni_1−x_Cr_x_ alloys, and the magnetic ordering temperature of these alloys decrease linearly with Cr doping^20^.

In spite of the extremely small magnetic moments shown in Fig. 3, the magnetic susceptibility data well above T_c_ are described by a Curie -Weiss law (see Supplemental information for more details) with relatively large effective magnetic moments per atom, M_eff_, as estimated from the Curie constant; M_eff_ = ≈2.2, 2, 1.3 and 0.8, μ_B_/atom for x = 0.5, 0.6, 0.7, 0.8. This is often the case for highly itinerant magnets such as ZrZn_2_^22^. A phenomenological measure of itinerant ferromagnetism was proposed by Rhodes and Wohlfarth^23^ who found that larger values of q_c_/M_o_ (or equivalently M_eff_/M_0_) termed the Rhodes-Wohlfarth (RW) parameter, corresponded to a more itinerant ferromagnet, where M_eff_^2^ = q_c_(q_c_ + 2). The RW parameters for the x = 0.5, 0.6, 0.7, and 0.8 alloys are ≈5, 8.5, 12, and 19. These large RW values are consistent with the presence of strong spin fluctuations^24^,^25^. A consequence of strongly itinerant ferromagnetism is that M_eff_ is determined by microscopic parameters of the band structure, and should not be regarded as implying a large local atomic magnetic moment^25^.

**Figure 4:**
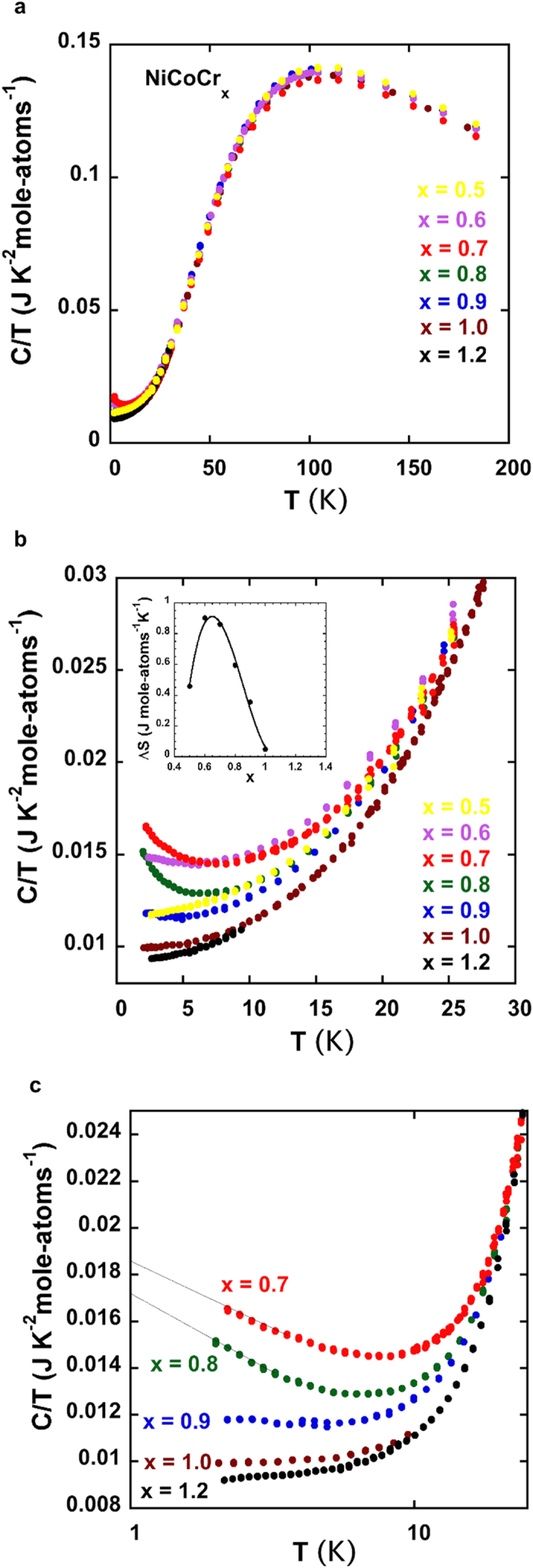
Heat capacity data. (**a**) Heat capacity divided by temperature (C/T) versus temperature for NiCoCr_x_ alloys for 0.5 < × < 1.2. (**b**) Same data as shown in (**a**) for T < 30 K. Inset shows the excess entropy, ΔS, versus Cr concentration x. ΔS is calculated with respect to the x = 1.2 heat capacity data. The maximum excess entropy occurs for x ≈ 0.65 and is about 15% of Rln2. (**c**) C/T data versus log T for 0.7 < × < 1.2. For x = 0.7, 0.8, the C/T data below 5 K are linear in -ln T down to our lowest measuring temperature of 2 K. The low temperature upturn in C/T can also be fit to a power law (Fig. S10).

Low temperature heat capacity measurements can probe some aspects of low energy magnetic or electronic excitations in a temperature regime where the contribution of the lattice is minimal. These have been frequently used to characterize, for example, non-Fermi liquid behavior near a magnetic instability in heavy fermion compounds like CeCu_5.9_Au_0.1_^26^,^27^. The heat capacity data from a series of NiCoCr_x_ alloys with 0.5 < × < 1.2 are shown in Fig. 4. Above 30 K the heat capacity, C, (Fig. 4a) of all of the alloys are the same within experimental error, which is approximately the size of the data points. Although magnetization data indicates magnetic order for alloys with x = 0.5, 0.6, 0.7 and 0.8, no anomaly is evident in the heat capacity data near T_c_, presumably because of the very small spontaneous moment (see Figs 3 and 4). A similar behavior was found for the weak ferromagnet ZrZn_2_ with T_c_ ≈ 28 K and M_0_ ≈ 0.16 μ_B_ per formula unit^28^. The heat capacity of the NiCoCr_x_ alloys are quite different below 20 K. The low temperature heat capacity data from the Pauli paramagnet NiCoCr_1.2_ appears to be a reasonable reference baseline for the other alloys. Standard C/T versus T^2^ of the NiCoCr_1.2_ data are linear below 10 K yielding a Debye temperature, Θ_D_, of 466 K and an electronic specific heat coefficient, γ, of 9.2 mJ K^−2^ mole-atoms^−1^. Both of these values are reasonable for this type of transition metal alloy^17^. As the Cr concentration is reduced below x = 1.2, there is a monotonic increase in C/T up to x = 0.7. For x = 0.7 and x = 0.8 C/T *increases* with cooling below 7 K. As x is reduced further away from the critical region toward the more ferromagnetic alloys (x = 0.5, 0.6), the low temperature C/T decreases in magnitude and begins to return to more normal metallic behavior. The excess low temperature entropy, ΔS, of all of the alloys with respect to the NiCoCr_1.2_ alloy, is estimated by integrating C/T from 0 to 30 K. A linear extrapolation of the C/T data was used below 2 K. The variation of ΔS with x is shown in the inset of Fig. 4b. The maximum in ΔS of about 0.9 J K^−1^ mole-atoms^−1^, occurs for x ≈ 0.65. The excess entropy at low temperatures is presumably associated with magnetic fluctuations. The amount of excess entropy is substantial when compared to the total magnetic entropy of a mole of spin ½ particles = Rln2 = 5.76 J K^−1^ mole-atoms^−1^. In Fig. 4c part of the data shown in 4b are plotted versus log T. Although we can only measure down to 2 K, the data below 5 K for x = 0.7 and 0.8 appears to be linear in lnT. A *–TlnT* contribution to the heat capacity has been reported by Lohneysen^26^,^27^ near the QCP of a heavy fermion alloy, CeCu_5.9_Au_0.1_, from 0.1 to 4 K and may be a common feature in many heavy fermion systems displaying non-Fermi-liquid behavior^26^,^27^. We note, however, that a power law (C ≈ T^α^), which has been proposed for some systems with strong disorder near a QCP (sometimes called Griffiths’ singularities), would also describe the low temperature heat capacity data for x = 0.7 and 0.8^10^,^11^,^29^ down to 2 K. A further test of magnetic quantum critical behavior in the NiCoCr_x_ alloys is the magnetic Grunheisen parameter^30^,^31^, which is defined as –(dM/dT)/C_p_. This quantity should diverge as the temperature approaches zero. From the magnetic and heat capacity data from the NiCoCr_x_ alloys (see supplemental Fig. S10) we estimate that the magnetic Grüneisen parameter diverges as T^−g^ with g = 2.8, 2.3, and 1.75 for x = 0.8, 0.9 and 1.0 respectively. We note that the divergence of the x = 0.8 Grüneisen parameter is very similar to that found for YFe_2_Al_10_^31^.

## Conclusions

We have shown that concentrated solid solution transition metal alloys (NiCoCr_x_) with a simple fcc structure display many of the transport, magnetic and thermodynamic signatures exhibited by more structurally complex compounds near a QCP^26^,^10^, such as a linear resistivity at low temperatures and a –TlnT or power law contribution to the heat capacity. The exponential dependence of the Curie temperature and saturation moment on composition near the QCP is perhaps the most striking and unique characteristic of these alloys. This class of alloys should provide a gateway to a clearer understanding of the role of disorder on the general behavior of matter near a ferromagnetic quantum critical point. Since these materials can be prepared as very large single crystals, powerful techniques such as inelastic neutron scattering and nuclear magnetic resonance can be applied to probe the dynamics of the electrons, spins, and lattice in these model materials.

Although mean field theories, such as KKR-CPA, capture much of the basic physics of these alloys, there are clearly large deviations between theory and experiment (e.g. see Fig. 3c) at low temperatures in the vicinity of the quantum critical region. It is hoped that the results reported in this article will stimulate the development of theories and scaling relationships similar to those proposed to explain the physics of the f-electron compounds near a QCP^26^,^27^.

## Methods

Polycrystalline samples of NiCoCr_x_ are prepared by arc-melting appropriate mixtures of the elements in an argon atmosphere. Each sample is melted and flipped a minimum of five times to insure complete mixing of the three elements. The well-mixed alloy is then dropcast into a 2 mm diameter rod, which is then polished to the desired shape. For resistivity measurements each alloy is polished to a bar with typical dimensions 10 × 0.3 × 0.3 mm^3^ or for Hall measurements to about 10 × 1 × 0.15 mm^3^. Single crystals of NiCoCr and NiCo are grown from the well-mixed polycrystalline alloys using a floating zone furnace. We found that the magnetic, transport and heat capacity data are virtually identical between the polycrystalline and single crystal samples of NiCoCr (see supplemental) and hence only data from polycrystalline samples are reported in the manuscript. Careful examinations of polished sections of each alloy using scanning electron microscopy and energy dispersive x-rays confirmed a complete random solid solution, as found by previous work^6^. Resistivity, Hall and heat capacity data are collected using a Physical Property Measurement System (PPMS) using standard methods. Four to six electrical contacts (0.05 mm Pt wires) are spot-welded to each alloy. Magnetic measurements are made with a Magnetic Property Measurement System (MPMS). See Supplemental Information for more details.

## Additional Information

**How to cite this article**: Sales, B. C. *et al.* Quantum Critical Behavior in a Concentrated Ternary Solid Solution. *Sci. Rep.* **6**, 26179; doi: 10.1038/srep26179 (2016).

## Supplementary Material

Supplementary Information
