# Supplementary material for: Quantum Critical Behavior in a Concentrated Ternary Solid Solution
Source: Sci Rep. 2016 May 18;6:26179. doi: 10.1038/srep26179 (PMC4870641; doi:10.1038/srep26179)
Supplement: Supplementary Information [file srep26179-s1.pdf]

Supplementary Information

Quantum Critical Behavior in a Concentrated Ternary Solid Solution

Brian C. Sales, Ke Jin, Hongbin Bei, G. Malcolm Stocks, German D. Samolyuk, Andrew F. May, Michael A. McGuire

Materials Science and Technology Division, Oak Ridge National Laboratory

S1 Lattice Constants versus Composition

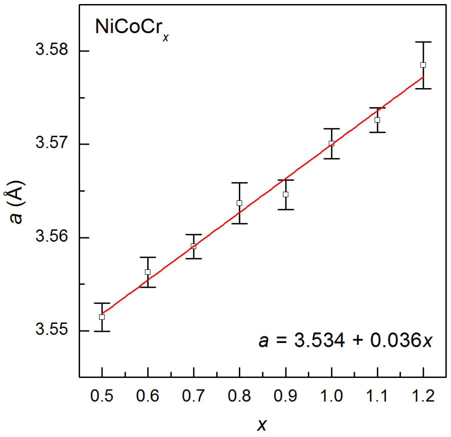

Figure S1. Lattice constants vs. Cr concentration x are determined from x-ray diffraction data from a flat polycrystalline surface. A PANalytical X'Pert Pro X-ray diffractometer with monochromatic Cu Kα_1 radiation was used for the measurements.

S2 Transport, magnetic and heat capacity measurements

Four wire resistivity measurements are made on rectangular bars of each alloy with approximate dimensions of 10 x 0.3 x 0.3 mm^3, using the resistivity option (AC mode, I= 4mA) of the Physical Property Measurement System (PPMS) from Quantum Design. Platinum wires with a diameter of 0.05 mm are spot-welded onto each sample. The contact resistance for all leads is less than 0.2 Ohms. Transverse magnetoresitance measurements are made on the same bars with the magnetic field perpendicular to the current I. Hall measurements were made on samples with typical dimensions of 10 x 1 x 0.15 mm^3 using four spot-welded leads in the standard Hall geometry.[1] Even with applied fields of up to 10 T, the Hall voltage is small for these alloys, yielding an approximate carrier concentration of ≈9 x 10^{22} holes/cm^3.

Magnetic data are taken with the Magnetic Property Measurement System (MPMS) from Quantum Design, which is a SQUID magnetometer. Magnetic moment data are collected as a function of temperature (2-400 K) in fixed applied fields of 0.01 to 1 Tesla, using both field cooled (FC) and zero-field-cooled (ZFC) protocols. Magnetic moment data (magnetization curves) are also collected at fixed temperatures with applied fields between 0 and 5 Tesla.

Heat capacity data are collected with the PPMS heat capacity option using the conventional relaxation method employing small heat pulses with the sample temperature rise restricted to 2% of the sample temperature.

S3 Average grain size and chemical homogeneity

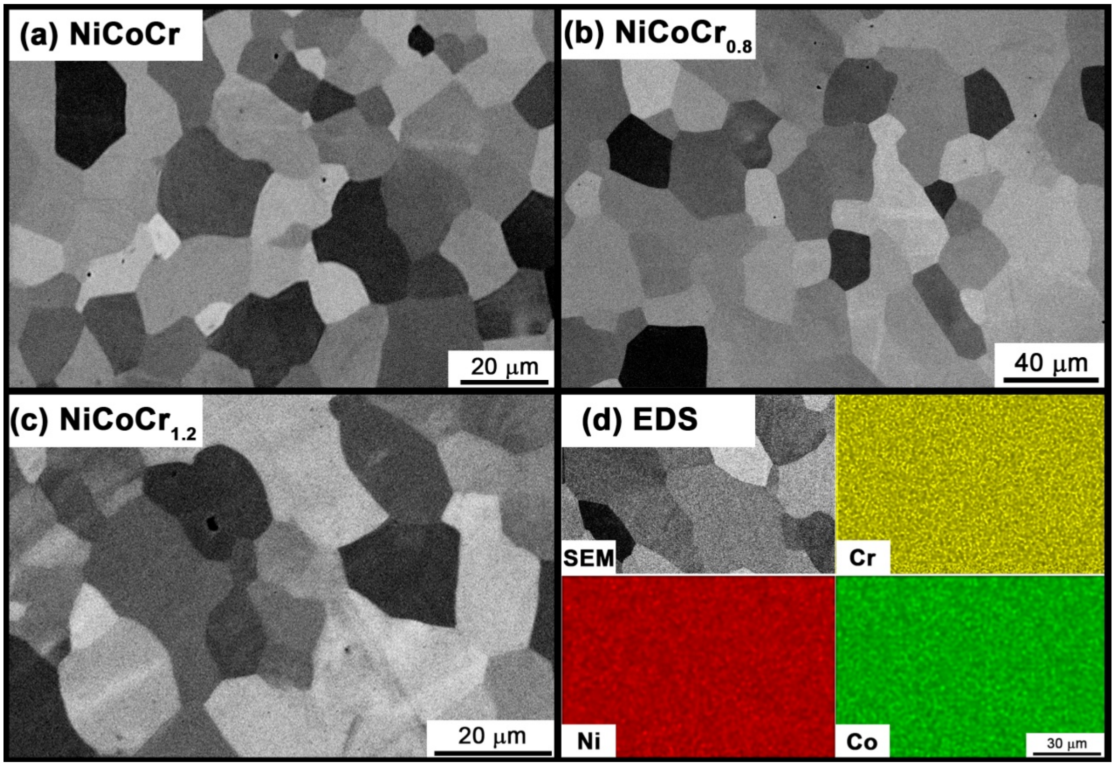

Figure S2. Microstructures and composition mapping of polycrystalline $NiCoCr_X$ alloys. (a-c) Electron backscatter images show the grain structures of NiCoCr, $NiCoCr_{0.8}$ and $NiCoCr_{1.2}$, respectively. (d) EDS mapping shows the homogeneous distributions of elemental Cr, Ni and Co in the NiCoCr polycrystalline alloys. All three alloys exhibit roughly equiaxed grains, with average grain sizes of 12 μm for NiCoCr, 16 μm for $NiCoCr_{1.2}$ and 26 μm for $NiCoCr_{0.8}$, respectively.

S4. Comparison of Polycrystalline NiCoCr vs Single Crystal NiCoCr Transport Data

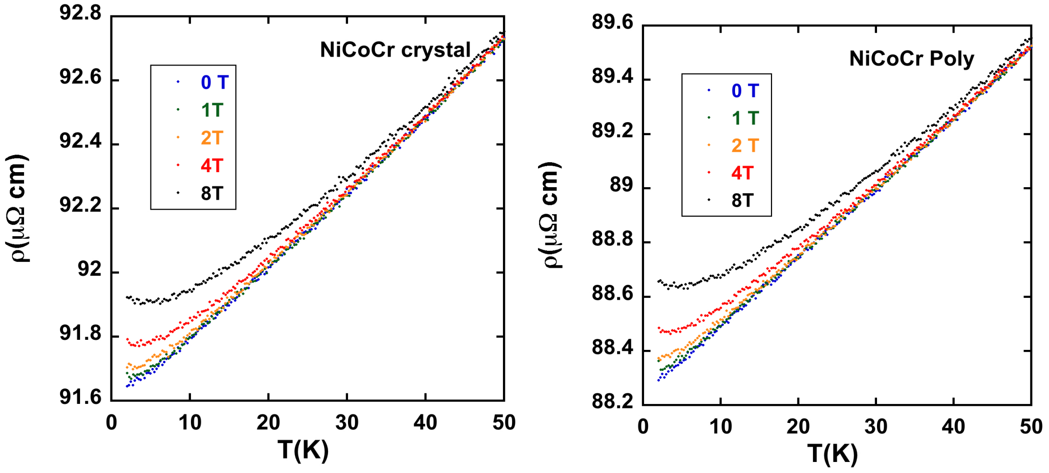

Figure S3. Resistivity of a NiCoCr single crystal (left) and a polycrystalline NiCoCr sample (right). The magnetic field is applied perpendicular to the current. The absolute values of the resistivity are only accurate to about 5% with most of the error determined by the measured distance between the voltage leads and the measured cross section area perpendicular to the current.

S5 Resistivity Data for other Cr Concentrations

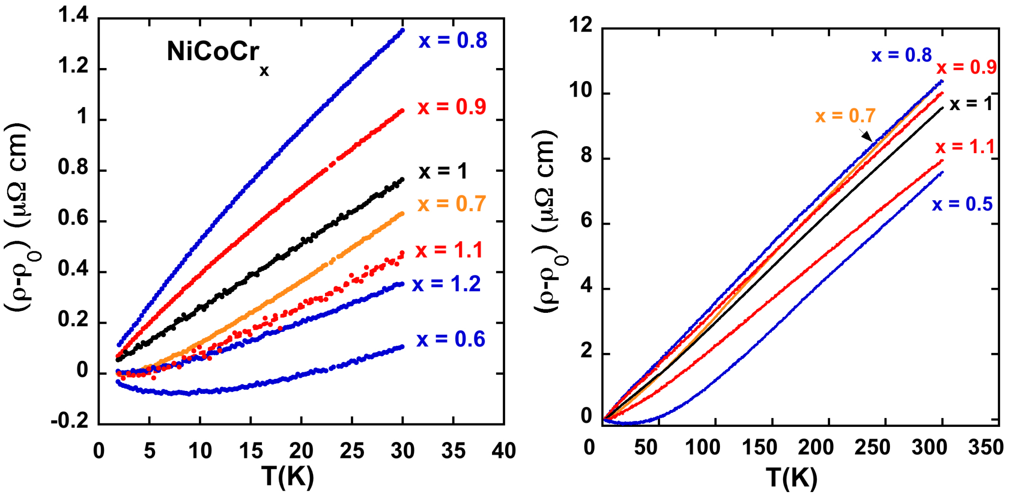

Figure S4 Resistivity of several polycrystalline $NiCoCr_x$ alloys (left) for temperatures between 2 and 30 K and (right) for temperatures between 2 and 300 K.

S6 Magnetoresistance Data for other Cr Concentrations

The low temperature tranverse magnetoresistance data from of *all* of the $NiCoCr_x$ alloys are close to linear in **H**, even for samples with compositions outside of the critical region. Since there is no indication of linear Dirac bands from electronic structure calculations (**see S10**), the linear magnetoresistance is likely due to the chemical disorder present in these alloys. [2,3]

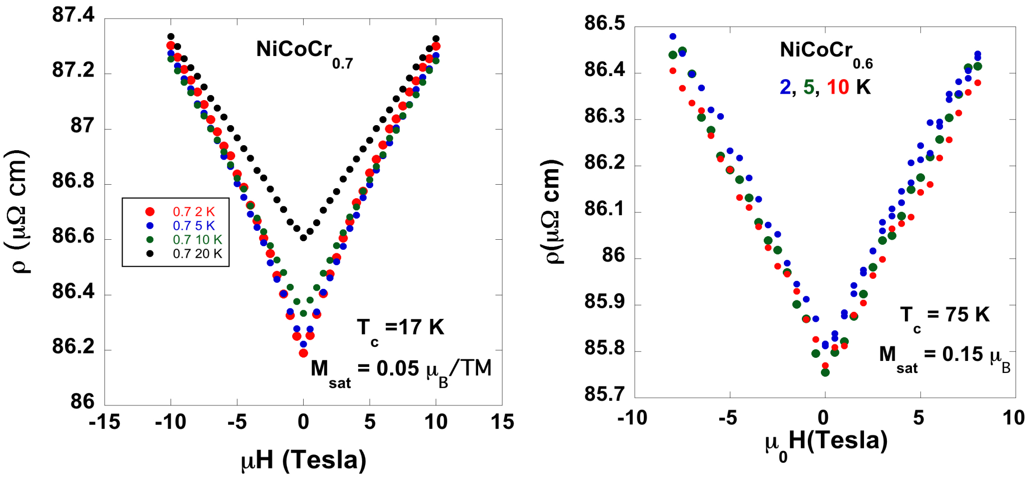

Figure S5. Transverse magnetoresistance for two of the weak itinerant ferromagnets $NiCoCr_{0.7}$ (left panel)and $NiCoCr_{0.6}$ (right panel)

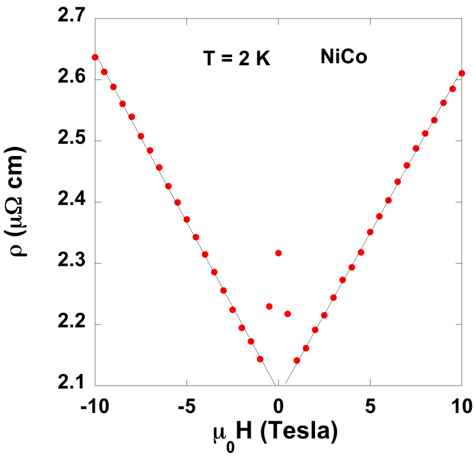

Figure S6. Transverse magnetoresistance for a NiCo single crystal (no Chromium) at 2 K. For low magnetic fields the resistance initially decreases as the ferromagnetic domains align, but for fields greater than 1 Tesla, the magnetoresistance is surprisingly linear.

S7 Determination of Curie Temperature

The Curie temperature, T_c, is determined by Arrott plots (M^2 vs H/M) at fixed temperatures and by extrapolation of plots of M^2 vs T with $\mu_0 H$ = 0.01 Tesla for temperatures below T_c.

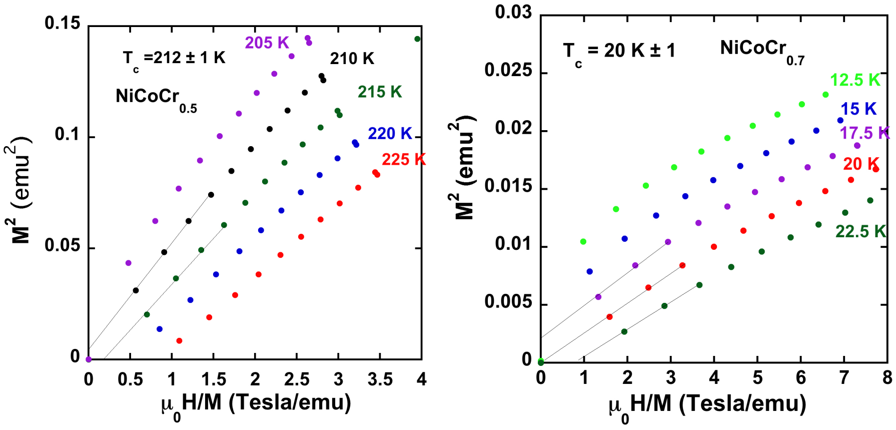

Figure S7. Arrott plot determination of Curie temperatures for $NiCoCr_{0.5}$ (left panel) and $NiCoCr_{0.7}$ (right panel).

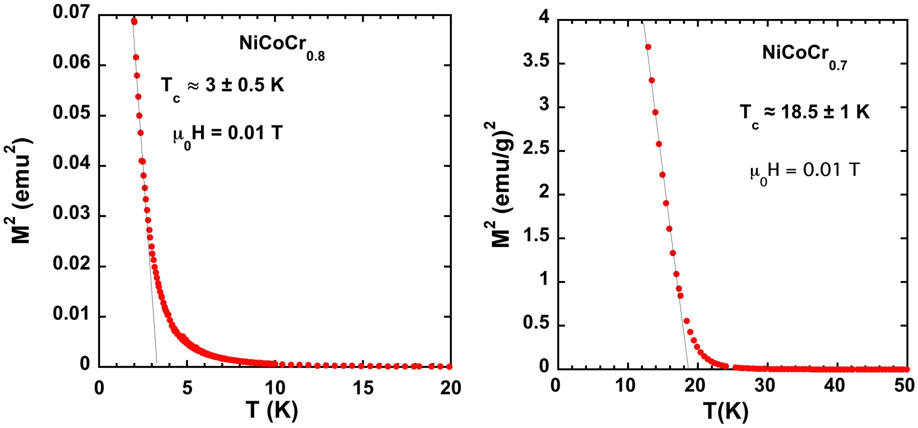

Figure S8 Determination of Curie temperature for $NiCoCr_{0.8}$ (left panel) and $NiCoCr_{0.7}$ (right panel) from M^2 vs T fits just below T_c. (This assumes a mean field exponent) We note that for $NiCoCr_{0.8}$ (left panel), an Arrott plot analysis of data from this sample yields a Curie temperature below 2K (our lowest measuring temperature) as compared to the T_c value of 3 K estimated in the Figure. This indicates that $NiCoCr_{0.8}$ is very close to the composition where ferromagnetism completely disappears.

S8. Determination of M_{eff} from Curie-Weiss plots well-above T_c (> ≈ $2T_c$)

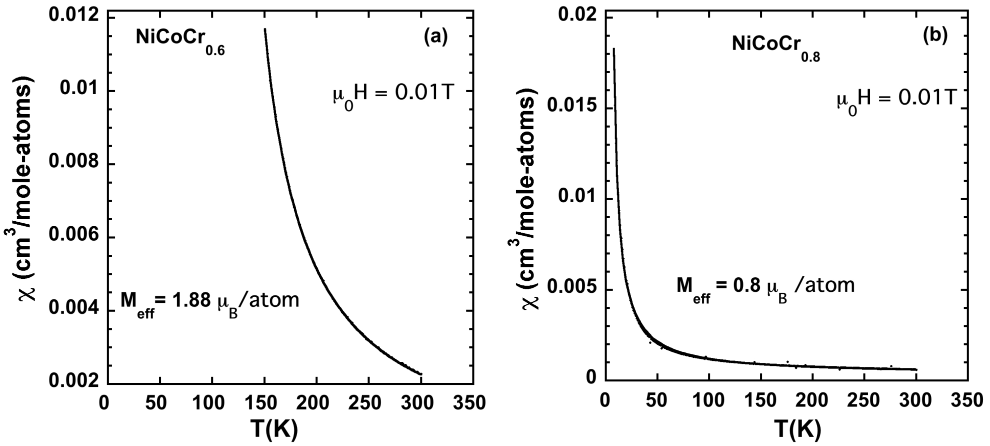

Figure S9. Curie-Weiss fits to $NiCoCr_{0.6}$ (left panel) and $NiCoCr_{0.8}$ (right panel) data at temperatures from $2T_c$ to 300 K. As noted in the main text, for a weak itinerant ferromagnet the values of M_{eff} should not be regarded as implying a large local magnetic moment on each atom but rather the values of M_{eff} are determined by microscopic parameters of the band structure.

S9 Estimation of the Magnetic Grüneisen Parameter

The magnetic Grüneisen parameter is defined as $(dM/dT)_H/C_p$ and has been shown to diverge at a magnetic quantum critical point as the temperature goes to zero. [4,5] For $NiCoCr_x$ alloys with x= 0.8, 0.9 and 1.0 it can be estimated from the low field (100 Oe) magnetization data, and the zero field heat capacity data shown below in **Fig. S10** .

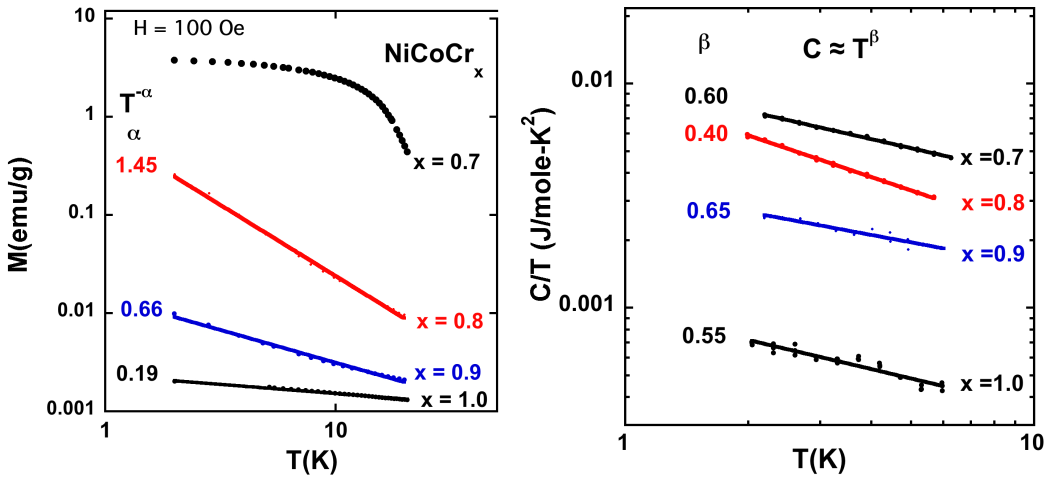

Figure S10 (left)Power law fit to M(T) data below 20 K. For Cr concentrations of x= 0.8, 0.9, and 1.0, the data are well described by a power law. (right) power law fit to excess heat capacity data below 6 K (The data from the $NiCoCr_{1.2}$ alloy was subtracted to remove any phonon contribution) . The exponents for each

composition are noted in the figure. From these data the magnetic Grüneisen parameter diverges as $T^{-\gamma}$ with γ = 2.8, 2.3, and 1.75, for x= 0.8, 0.9 and 1.0 respectively. We note that the divergence of the x=0.8 Grüneisen parameter is very similar to that found for YFe_2Al_{10}.[5]

S10. Mean field KKR-CPA calculations: methods and results

Methods: Electronic structure calculations in substitutional alloys.

The $NiCoCr_x$ electronic structure calculations were performed for an ideal fcc structure using the experimentally determined lattice parameters: 3.53 and 3.57 Å for NiCo and NiCoCr respectively. For the compositions x between 0 and 1 the lattice parameter was estimated according to Vegard's law. The residual resistivity was calculated using formalism based on one-electron Kubo formula[12-14] within fully relativistic Korringa-Kohn-Rostoker[4] Coherent-Potential-Approximation[7,8] (KKR-CPA) method, as implemented in SPRKKR code[7,8]. The KKR-CPA method describes the effects of chemical disorder on the electronic states, and delivers the configurationally averaged properties, for example, electronic structure, magnetic structure and transport properties. The calculations have been performed using the local spin density approximation LDA to density functional theory (DFT) and the Vosko, Wilk and Nusair parameterization of the exchange-correlation function[11]. The energy integration was executed over 32 points in complex energies plane. The Brillouin zone (BZ) summations over special k-points were over 28×28×28 k-points mesh during the density functional theory self-consistency cycle and 78×78×78 k-point mesh for the residual resistivity calculation. An angular momentum cutoff of 4 was used in the solution of the multiple-scattering equations.

Calculations of the configurationally averaged densities of states and Bloch spectral function along high-symmetry directions for pure nickel and the substitutionally disordered equiatomic NiCo and NiCoCr and NiCoFe alloys were performed using the ab initio KKR-CPA electronic structure method as implemented in the Hutsepot code. An angular momentum cutoff of 3 was used in the solution of the multiple-scattering equations. The KKR-CPA scattering-path matrix was calculated in reciprocal (k) space using a 20×20×20 k-point mesh during the density functional theory self-consistency cycle and 50×50×50 k-point mesh for the DOS calculation. The DOS and BSF were calculated at energy of 0.001 Rydberg off into the upper half of the complex plane, which gives rise to the slight broadening of the BSF for pure nickel.

Results and discussion:

To characterize the effects of chemical disorder on the electronic and magnetic properties, we performed electron structure calculations using ab initio Korringa–Kohn–Rostoker coherent-potential-approximation (KKR-CPA) method. The method, implemented within density functional theory, provides an ab initio theoretical description of the effects of disorder on the underlying electronic structure. An important aspect of the KKR-CPA is that it is specifically formulated to calculate the configurationally averaged electronic structure, including local and total densities of states (DOS) and magnetic moments within a single-site (or mean-field) theory. Moreover, the KKR-CPA has also been extended to calculate many other properties, including electron transport. Here it is worth noting that it is the configurationally averaged properties of random solid solutions that are actually measured by experiments. Figures S11, S12, and S13 show the Bloch spectral function (BSF), which is a generalization of the band structure of an ordered system to include disorder, and configurationally averaged DOS of NiCo, NiCoFe and NiCoCr, respectively. In these figures, the left and right-hand panels show the contributions from the spin-down (minority) and spin-up (majority) electrons the BSF and DOS, respectively. As can be seen from the BSF plots, for NiCo (Fig. S11) and NiCoFe (Fig. S12) the d-band smearing is largely limited to minority states; whereas in NiCoCr (Fig. S13), both minority and majority states are smeared, giving rise to the much larger overall smearing. The Fermi energy wave vector broadening of the BSF is related to the inverse of the electron mean free path. At the Fermi energy, k-space smearing implies a decrease in electron mean free path. No (or little) smearing implies an infinite (long) mean free path, whereas uniform smearing throughout the Brillouin zone implies a mean free path on the order of the interatomic spacing. While the electron mean free path for the NiCo and NiCoFe alloys (Fig. S11, S12) is short for the minority spin electrons, it is large for the majority spin electrons thereby providing a short circuit and an overall low resistivity. On the contrary, for the NiCoCr alloy (Fig. S13), both channels are broadened, particularly near the Fermi energy, implying a short mean free path in both spin channels. The consequently strong electron–electron scattering can lead to a high residual resistivity. Our calculated residual resistivity results are in full agreement with this qualitative explanation (Table S1). Thus, the resistivity in $NiCoCr_x$ monotonically increase from 2.28 to 70.0 μΩcm with composition x increase from 0 to 1.1.

Table S1. Average moment in Bohr magnetons on Ni, Co, and Cr atoms in $NiCoCr_x$ alloys as a function of Cr content x, as determined from KKR-CPA calculations. The average moment per atom in each alloy is also shown, as is the calculated residual resistivity in μΩcm for selected alloys. The results are calculated using SPR-KKR code[9,10]. A negative magnetic moment for Cr indicates it is antiparallel to the Ni and Co moments.

x	M_{Ni}	M_{Co}	M_{Cr}	M_{av}	ρ_0
0.0	0.62	1.59	-1.69	1.11	2.28
0.1	0.53	1.49	-1.17	0.91	41.91
0.3	0.41	1.34	-0.53	0.69	56.39
0.4	0.35	1.25	-0.40	0.60	59.90
0.5	0.31	1.17	-0.32	0.53	62.59
0.7	0.22	0.98	-0.21	0.39	66.23
0.8	0.18	0.88	-0.18	0.33	67.46
0.9	0.15	0.78	-0.14	0.27	68.45
1.0	0.11	0.61	-0.10	0.20	69.50
1.1	0.09	0.52	-0.08	0.16	70.00

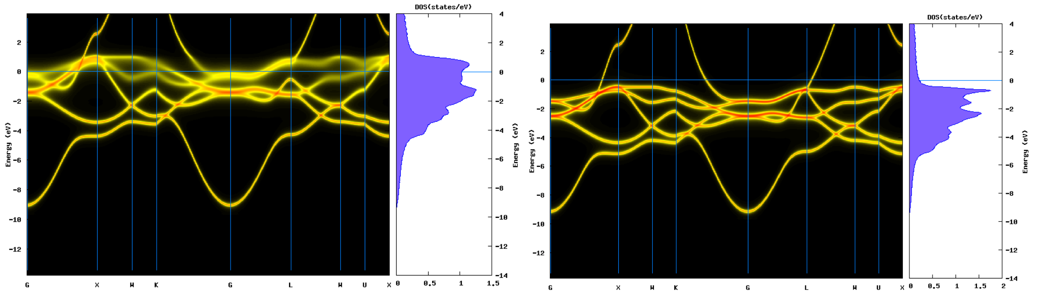

Figure S11. Band structure function and corresponding density of states NiCo minority (left) and majority (right) spin channel

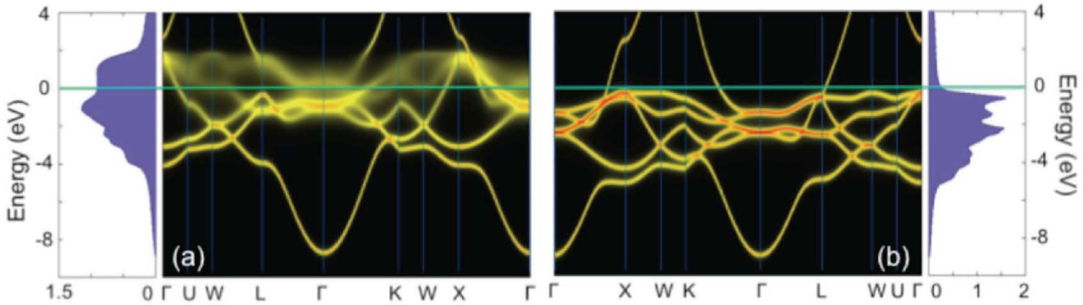

Figure S12. Band structure function and corresponding density of states in NiCoFe a) minority spin channel, b) majority spin channel.

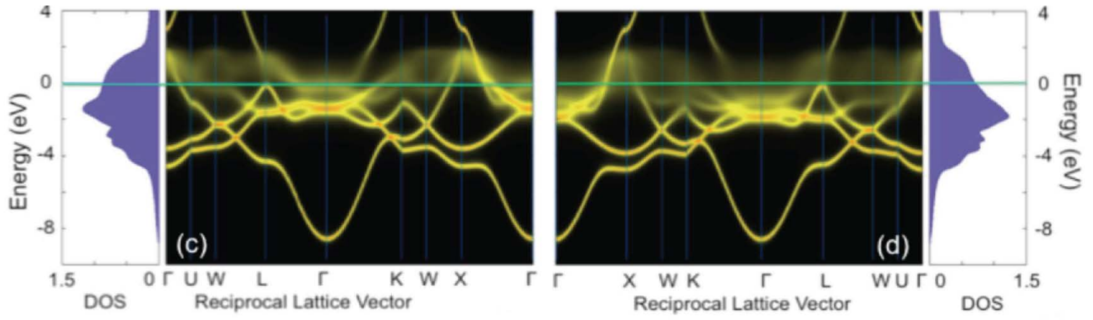

Figure S13. Band structure function and corresponding density of states in NiCoCr, c) minority spin channel, d) majority spin channel.

References

1. Sales, B. C., Jin, R.Y., Mandrus, D. Orientation dependence of the anomalous Hall resistivity in single crystals of $Yb_{14}MnSb_{11}$, *Phys. Rev. B.* **77**, 024409-1-9 (2008).

2. Xu, R., Husmann, A., Rosenbaum, T. F., Saboungi, M. L., Enderby, J. E., Littlewood, P. B. Large magnetoresistance in non-magnetic silver chalcogenides. *Nature* **390**, 57-60 (1997).

3. Song, J. C. W., Refael, G., Lee P. A. Linear magnetoresistance in metals: Guiding center diffusion in a smooth random potential. *Phys. Rev. B.* **92**, 180204-1-5 (2015).

4. Zhu, L, Garst, M., Rosch, A., Si, Q Universally Diverging Grüneisen Parameter and the Magnetocaloric Effect Close to Quantum Critical Points, *Phys. Rev. Lett.* **91**, 066404 (2003).

5. Wu, L. S., Kim, M. S., Park, K., Tsvelik, A. M., Aronson, M. C. Quantum critical fluctuations in layered YFe_2Al_{10}, *PNAS* **111**, 14086 (2014).

4. Korringa, J. On the calculation of the energy of a Bloch wave in a metal, Physica 13, 392 (1947). Kohn, W., Rostoker, N., Solution of the Schrödinger Equation in Periodic Lattices with an Application to Metallic Lithium, *Phys.Rev.* **94**, 1111 (1954).

5. Velický, B., Kirkpatrick, S. and Ehrenreich, H., Single-Site Approximations in the Electronic Theory of Simple Binary Alloys, *Phys. Rev.* **175**, 747 (1968).

6. Stocks, G.M., Temmerman, W.N., Gyorffy, B.L. Complete Solution of the Korringa-Kohn-Rostoker Coherent-Potential-Approximation Equations: Cu-Ni Alloys, *Phys. Rev. Lett.* **41**, 339 (1978).

7. The Munich SPR-KKR package, version 6.3, H. Ebert et al, http://ebert.cup.uni-muenchen.de/SPRKKR.

8. Ebert, H., Ködderitzsch, D., and Minár, J. Calculating condensed matter properties using the KKR-Greens's function method - recent developments and applications, *Rep. Prog. Phys.* **74**, 96501 (2011).

9. Vosko, S.H., Wilk, L., and Nusair, M. Accurate spin-dependent electron liquid correlation energies for local spin density calculations: a critical analysis, *Can. J. Phys.* **58**, 1200 (1980).

10. Butler, W. H. Theory of electronic transport in random alloys: Korringa-Kohn-Rostoker coherent-potential approximation. *Phys. Rev. B* **31**, 3260–3277 (1985).

11. Swihart, J. C., Butler, W. H., Stocks, G. M., Nicholson, D. M., Ward, R. C. First-principles calculation of the residual electrical resistivity of random alloys. Phys. Rev. Lett. **57**, 1181–1184 (1986).

12. Ködderitzsch, D., Lowitzer, S., Staunton, J. B., Ebert, H. Electronic and transport properties of disordered transition-metal alloys. *Phys. Status Solidi B* **248**, 2248–2265 (2011).
